# Assessing Human Embryonic Stem Cell-Derived Dopaminergic Neuron Progenitor Transplants Using Non-invasive Imaging Techniques

**DOI:** 10.1007/s11307-020-01499-4

**Published:** 2020-05-06

**Authors:** M. Mousavinejad, S. Skidmore, F. G. Barone, P. Tyers, V. Pisupati, H. Poptani, A. Plagge, R. A. Barker, P. Murray, A. Taylor, C. J. Hill

**Affiliations:** 1 Department of Cellular and Molecular Physiology, Institute of Translational Medicine, University of Liverpool, Liverpool, L69 3BX UK; 2 WT-MRC Cambridge Stem Cell Institute, University of Cambridge, Cambridge, UK; 3 John van Geest Centre for Brain Repair & Department of Neurology, Department of Clinical Neurosciences, University of Cambridge, Cambridge, UK; 4 Centre for Women’s Health Research, Department of Women’s and Children’s Health, Institute of Translational Medicine, University of Liverpool, Liverpool, L8 7SS UK

**Keywords:** Dopaminergic neuron progenitor cells, Human pluripotent stem cell, Parkinson’s disease, Non-invasive imaging, Bioluminescence, Magnetic resonance imaging

## Abstract

**Purpose:**

Human pluripotent stem cell (hPSC)-derived dopaminergic neuron progenitor cells (DAPCs) are a potential therapy for Parkinson’s disease (PD). However, their intracranial administration raises safety concerns including uncontrolled proliferation, migration and inflammation. Here, we apply a bimodal imaging approach to investigate the fate of DAPC transplants in the rat striatum.

**Procedures:**

DAPCs co-expressing luciferase and ZsGreen or labelled with micron-sized particles of iron oxide (MPIOs) were transplanted in the striatum of RNU rats (*n* = 6 per group). DAPCs were tracked *in vivo* using bioluminescence and magnetic resonance (MR) imaging modalities.

**Results:**

Transgene silencing in differentiating DAPCs accompanied with signal attenuation due to animal growth rendered the bioluminescence undetectable by week 2 post intrastriatal transplantation. However, MR imaging of MPIO-labelled DAPCs showed that transplanted cells remained at the site of injection for over 120 days. Post-mortem histological analysis of DAPC transplants demonstrated that labelling with either luciferase/ZsGreen or MPIOs did not affect the ability of cells to differentiate into mature dopaminergic neurons. Importantly, labelled cells did not elicit increased glial reactivity compared to non-labelled cells.

**Conclusions:**

In summary, our findings support the transplantation of hPSC-derived DAPCs as a safe treatment for PD.

**Electronic supplementary material:**

The online version of this article (10.1007/s11307-020-01499-4) contains supplementary material, which is available to authorized users.

## Introduction

Parkinson’s disease (PD) is a neurodegenerative disease that results in part from the progressive loss of dopaminergic (DA) neurons in the substantia nigra. Several groups have shown that human pluripotent stem cell (hPSC)-derived dopaminergic neuron progenitor cells (DAPCs) can generate mature DA neurons and improve motor function following intrastriatal transplantation in animal models of PD [1, 2]. This has now evolved to the point that the first in human hPSC-based DA neural transplants are being undertaken or planned in patients with PD. However, prior to undertaking larger-scale clinical studies, animal experiments are needed to adequately assess the safety of the therapies. Key safety concerns with such therapies for PD and other central nervous system (CNS) disorders include the risk that the implanted cells could proliferate and form space-occupying masses and/or migrate to off-target sites within the CNS and/or induce major neuroinflammation [3]. In addition to considering the potential risks, it is also important to monitor the long-term viability and differentiation capacity of implanted cells, as to be effective, they must differentiate into the appropriate phenotype and persist in the brain.

An effective strategy for monitoring the proliferation, viability and localisation of implanted cells longitudinally is to employ a non-invasive imaging approach comprising different modalities, such as bioluminescence (BLI), magnetic resonance (MRI) and fluorescence imaging [4, 5]. BLI is the most sensitive live animal imaging technique, enabling relatively small numbers of transplanted cells to be detected [6]. This technique requires that the cells express a luciferase reporter, which means that a signal is only emitted if the cells are alive. An increase in BLI signal over time indicates cell proliferation and potential tumour formation whereas a loss of signal suggests that the cells are no longer viable. A drawback with BLI, however, is that spatial resolution is poor, which means that it cannot be used to determine the location of the implanted cells and/or any resultant masses within the brain. MR imaging, on the other hand, has a very high spatial resolution and can accurately map the position of intracranial lesions [7]. Moreover, by labelling cells prior to administration with an appropriate contrast agent, such as iron oxide particles [8] or ^19^F-based tracking agents [9], MRI can be used to plot the biodistribution of the cells over time.

Hoehn and co-workers have shown previously that BLI and MR imaging can be used to monitor the viability and intracranial biodistribution of human embryonic stem cell (hESC)-derived neural stem cells following implantation into the mouse striatum [10]. To the best of our knowledge, this bimodal approach has not previously been used to track the tumourigenicity, viability and biodistribution of hESC-derived DAPCs, following intrastriatal administration into the rat brain. A key aim of this study, therefore, was to assess the potential of this bimodal BLI/MR strategy to track hESC-derived DAPCs *in vivo*. In addition to evaluating the effectiveness of the imaging modalities themselves, we also investigated whether the labels used for tracking (*i.e.* a firefly luciferase, Fluc-ZsGreen bicistronic vector for BLI and iron oxide particles for MR imaging [4, 11]) affected the differentiation potential of the cells and/or their immunogenicity following implantation into the rat striatum.

## Materials and Methods

### hESC Culture and Maintenance

The clinical-grade RC17 hESC line was obtained from Roslin Cells Ltd., UK. Cells were expanded on laminin 521 (0.5 μg/cm^2^) (Biolamina) in iPS-Brew XF (StemMACS™). Cells were passaged as small clumps using Versene, a non-enzymatic cell dissociation reagent (ThermoFisher Scientific), and 10 μM of Rho kinase (Rock) inhibitor Y-27632 dihydrochloride (StemMACS, Miltenyi) was added to the medium for the first 24 h after plating. The medium was changed daily, and cells were maintained at 37 °C under 5 % CO_2_.

### Generation of hESC Reporter Line and Labelling with Iron Oxide Particles

RC17 cells were transduced with a lentiviral vector encoding for the bicistronic expression of the codon-optimised firefly luciferase (luc2) and ZsGreen (*via* an IRES link) under the constitutive promoter elongation factor-α (EF1α). The vector plasmid was a gift from Bryan Welm (Addgene plasmid #39196), and the production and titration of viral particles was carried out using established protocols [11]. In order to transduce the hESCs, colonies of undifferentiated RC17 cells were dissociated into very small clumps consisting of about 10–15 cells using Versene for 5 min. After centrifugation, the cells were counted and seeded onto laminin 521 at a density of approximately 2.5 × 10^4^ cells/cm^2^ in the presence of 10 μM Y-27632. Cells were incubated overnight and transduced on the following day with 25 × 10^4^ viral particles (multiplicity of infection of approximately 5) in the presence of polybrene (10 μg/ml). After 24 h, the medium was replaced, and the cells were expanded for 4 days prior to sorting for ZsGreen expression with a BD FACSAria (BD Biosciences) flow sorter. The Fluc-ZsGreen^+^ cells were collected in iPS-Brew culture medium supplemented with 10 μM Y-27632, seeded on laminin 521 and expanded for subsequent experiments. To assess bioluminescence activity, cells were plated at different densities in black 96-well plates (Thermo Scientific), allowed to settle for 2–4 h and then incubated with medium containing D-luciferin (150 μg/ml, Promega) prior to data acquisition with an IVIS spectrum system (Perkin Elmer).

Micron-sized particles of iron oxide (MPIO) were used as a label for MR detection of DAPCs. Suncoast Yellow MPIOs (Bangs Beads, 1.63 μm nominal diameter, Bangs Laboratories, Inc.) were added directly to the DAPC’s cell culture medium at a concentration of approximately 1500 particles/μl for 24 h. After the labelling period, cells were carefully washed with PBS to remove unbound particles, harvested and then used for *in vivo* studies. The extent of MPIO labelling was assessed with a FACSCalibur (BD Biosciences) flow cytometer.

### Differentiation into Neural Precursors and Mature Neurons

RC17 cells were differentiated towards mesencephalic DAPCs or terminally differentiated into mature DA neurons as previously described [12]. In brief, DAPCs are obtained after neuralisation, patterning and expansion of the cells for a period of 16 days whereas DA neurons are obtained *via* the maturation of DAPCs for 34 days. Correct caudalization of progenitors towards a midbrain fate was achieved using 0.9 μM GSK3 inhibitor (CHIR99021).

### Cell Implantation and *In Vivo* Imaging

RNU rats (males, 5–6 weeks old) were purchased from Charles River and housed in individually ventilated cages under a 12-h light/dark cycle with *ad libitum* access to standard food and water. All animal experiments were performed under a licence granted through the UK Animals (Scientific Procedures) Act 1986 and were approved by the University of Liverpool ethics committee. All applicable institutional and/or national guidelines for the care and use of animals were followed. All procedures (surgical administration of cells and imaging) were carried out under isoflurane anaesthesia.

Single-cell suspensions prepared in Hanks’ Balanced Salt Solution were implanted stereotactically into the left and right hemispheres of the rats’ brains. Using the bregma as a reference, the skull was drilled at 0 mm anteroposterior and ±1.5 mm mediolateral, with each hemisphere receiving two deposits of cells at a depth of −5.0 and −4.3 mm from the dura. Each deposit contained 75 × 10^3^ cells in 0.75 μl of PBS, delivered with a microsyringe connected to an infusion pump. The rats were divided into three different experimental groups as outlined in Table 1.

**Table 1.** Description of the experimental groups

Group	Cells implanted in the left hemisphere	Cells implanted in the right hemisphere	Number of animals	Endpoint
1	Undifferentiated hESCs	Undifferentiated hESCs (Fluc-ZsGreen^+^)	3	Day 27
2	DAPCs	DAPCs (Fluc-ZsGreen^+^)	6	Day 91
3	DAPCs	DAPCs (MPIO-labelled)	6	Day 127

BLI was carried out with an IVIS spectrum system. After inducing anaesthesia, the rats’ heads were shaved and the animals received an intraperitoneal injection of luciferin at a dose of 150 mg/kg body weight. Data were acquired 20 min post administration of the substrate with a field of view B (6.5 cm), medium binning, f-stop 1 and exposure time calculated automatically by the acquisition software, up to a maximum of 5 min. All bioluminescence data were normalised to the acquisition conditions and are displayed as radiance (photons/s/cm^2^/str).

MRI data were acquired with a Bruker Avance III console interfaced to a 9.4T magnet system (Bruker Biospec 94/20 USR). RF excitation was achieved with an 86-mm resonator and signal detection with a four-channel phased array receive-only rat brain coil. Once the injection site was located using scout images, higher resolution images were acquired with rapid acquisition with relaxation enhancement (RARE) sequence. Following are the parameters used: echo time (TE) = 38 ms, repetition time (TR) = 2700 ms, RARE factor = 8, number of excitations (NEX) = 8, field of view (FOV) = 35 × 35 mm, matrix size = 350 × 350 pixels, slices = 20, slice thickness = 500 μm.

At the experimental endpoint, the rats received an overdose of pentobarbital and were perfused transcardially with PBS followed by 4 % formaldehyde. The brains were harvested, postfixed with 4 % formaldehyde, equilibrated in 30 % sucrose and cryosectioned for microscopy analysis.

### RT-qPCR

Cells were washed twice with PBS, and a minimum of 5 × 10^5^ cells were lysed with TRI Reagent (Sigma). Total RNA was extracted according to the manufacturer’s protocol, and a NanoDrop was used to determine RNA concentration. To synthesise cDNA, RNA was treated with DNase1 and reverse transcribed using random hexamers (Qiagen) and Superscript III reverse transcriptase (Invitrogen). PCR was performed on a CFX Connect system (Bio-Rad) using SYBR Green JumpStart Taq Ready Mix (Sigma). The genes OTX2, FOXA2 and LMX1A were measured to assess differentiation into DAPCs, with GAPDH being used as a housekeeping gene. Undifferentiated hESCs were used as a control. Relative expression levels of target genes between control and experimental samples were calculated using the 2^−ΔΔCt^ method [13]. Primer sequences are shown in ESM Table 1.

### Immunostaining and Histology

Cells were fixed with 4 % formaldehyde for 20 min, permeabilised with 0.1 % Triton X-100 for 20 min and blocked with 1 % bovine serum albumin (BSA) for 30 min. Cryosections (10 μm) from fixed tissues were permeabilised and blocked as described above. Primary antibodies were diluted in 1:1 Triton X-100:BSA according to the dilution factors in ESM Table 2 and incubated for 24 h at 4 °C. Secondary antibodies were diluted 1:1000 in 1:1 Triton X-100:BSA and incubated for 2 h at room temperature. For immunofluorescence, cells were counterstained with DAPI. Images were acquired on a 3i spinning disk confocal microscope CSU-X1 (Intelligent Imaging Innovations) and processed with ImageJ [14]. For immunohistochemistry, tissue sections were incubated with 1.4 mM 3,3′-diaminobenzidine (DAB) and 0.01 % hydrogen peroxide for 15 min, and images were acquired with a Leica DM IL microscope.

## Results

### hESC Labelling Does Not Negatively Impact on Differentiation Towards DAPCs or Mature Dopaminergic Neurons *In Vitro*

**Fig. 1.**
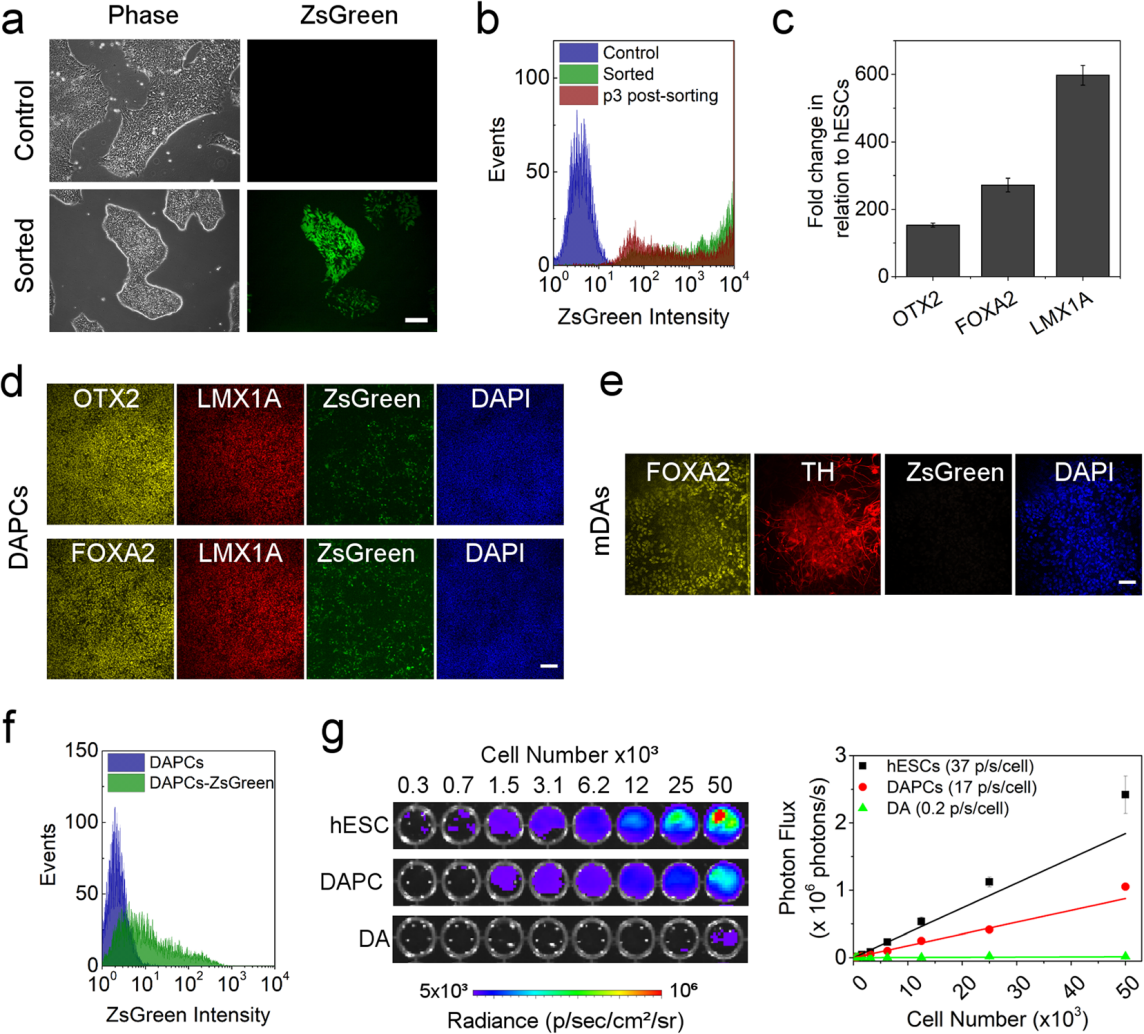
**Effect and stability of the Fluc-ZsGreen reporter gene in hESCs. a** Phase contrast and fluorescence microscopy of the control and Fluc-ZsGreen^+^ hESCs. Cells were imaged three passages post sorting. **b** ZsGreen expression, as measured *via* flow cytometry, of the control and sorted hESCs. The green fluorescence of the sorted cells is stable for several passages. **c** Expression of OTX2, FOXA2 and LMX1A in DAPCs obtained from Fluc-ZsGreen^+^ hESCs. **d**, **e** Fluorescence microscopy of DAPCs and DAs obtained from Fluc-ZsGreen^+^ hESCs (differentiation days 15 and 50, respectively). Cells were immunostained for OTX2, FOXA2, LMX1A and TH. **f** Flow cytometry shows that differentiation into DAPCs reduces ZsGreen expression (approximately 47 % of the cells expressing the construct). **g** BLI of different numbers of Fluc-ZsGreen^+^ hESCs, DAPCs and DAs and the corresponding photon flux. Data are representative of three independent experiments. Error bars represent SD, and the solid line the linear fit of the data. Scale bars in micrographs correspond to 100 μm.

Flow cytometry analysis of RC17 hESCs 4 days after viral transduction showed that approximately 47 % of the population expressed the reporter gene ZsGreen (ESM Fig. 1a, b). After sorting, a pure population of hESCs expressing the reporter was obtained (Fig. 1a, b), herein defined as Fluc-ZsGreen^+^ hESCs. Fluc-ZsGreen^+^ hESCs maintained expression of ZsGreen over multiple passages and were morphologically indistinguishable from non-transduced cells (Fig. 1a, b). To assess whether the introduction of the reporter affected pluripotency, embryoid bodies (EB) were generated and immunostained for markers of embryonic germ layer derivatives. The presence of GATA6 (endoderm), Brachyury (mesoderm) and Nestin (ectoderm) confirmed that the Fluc-ZsGreen^+^ hESCs remained pluripotent (ESM Fig. 1c).

DAPCs were assessed for the co-expression of the key markers FOXA2, LMX1A and OTX2 on day 16 of differentiation. Quantification of mRNA *via* RT-qPCR revealed significant upregulation of all these markers (Fig. 1c), which was confirmed *via* immunofluorescence (Fig. 1d). However, fluorescence microscopy also revealed that not all cells expressed ZsGreen after differentiation into DAPCs (Fig. 1d). DAPCs were further differentiated into mature DA neurons and immunostained (differentiation day 50) to detect the classic DA neuron marker, tyrosine hydroxylase (TH). Immunofluorescence demonstrated that the transduced RC17-derived DA neurons expressed TH (Fig. 1e), but ZsGreen was no longer detectable at this differentiation stage.

Flow cytometric analysis of DAPCs showed that only 51 % of these cells expressed ZsGreen, implying a significant loss of reporter gene expression when compared to undifferentiated hESCs (Fig. 1f), and complete loss once the cells had matured to dopaminergic neurons (DAs) (Fig. 1e). Measurement of the light output (bioluminescence) revealed that the expression of luciferase corresponded to that of ZsGreen; that is, bioluminescence was strong before differentiation (37 p/s/cell), significantly reduced as cells differentiated towards DAPCs (17 p/s/cell) and extremely weak when they became mature DAs (<1 p/s/cell) (Fig. 1g).

Taken together, these results show that the introduction of the genetic reporter did not affect hESC pluripotency nor their ability to differentiate to DAPCs and DA neurons. However, reporter gene expression was progressively lost as the cells differentiated towards DA neurons. Despite the reduction in light emission in DAPCs, we reasoned that it would still be possible to detect them in rodents *in vivo*, enabling their tracking and assessment of viability/tumourigenicity in the early post-transplant period, but that it would not be possible to detect the mature DA neurons.

### *In Vivo* Imaging Reveals Absence of DAPC Tumourigenicity and Long-Term Intracranial Distribution

**Fig. 2.**
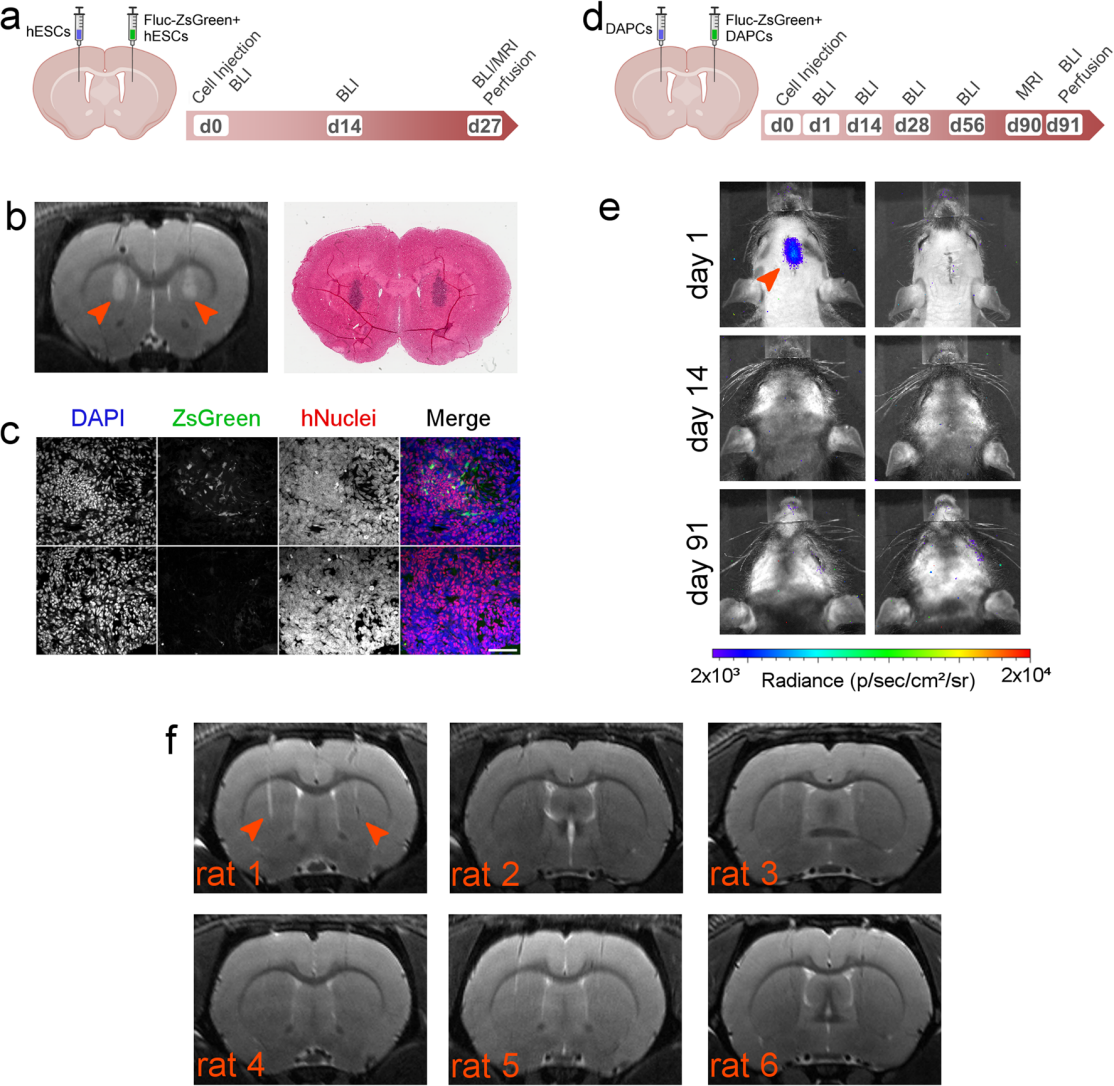
**Long-term fate of hESCs and DAPCs. a** Schematic of injection and experimental timeline of hESC administration and imaging. **b** Representative RARE MRI scan (day 27) and corresponding histological section (H&E staining) of a rat that received undifferentiated hESCs (left hemisphere: non-transduced, right hemisphere: Fluc-ZsGreen^+^). Both sides display a large area of hyperintense contrast at the injection site (arrowheads) which was confirmed to correspond to tightly packed cell nuclei *via* histology. **c** Fluorescence microscopy of areas of abnormal growth in the right hemisphere. In all cases, the growth corresponded to cells of human origin, as evidenced by expression of a human nuclear antigen. The level of ZsGreen expression was heterogeneous within the growths, with areas of strong expression (top) and areas where ZsGreen was lost (bottom). Scale bar = 50 μm. **d** Schematic of injection and experimental timeline of DAPC administration and imaging. **e** BLI of two of the six rats that received DAPCs as imaged on days 1, 14 and 91. Most, but not all, rats displayed a signal on the injection day, but this was lost by day 14, and no signal was seen at any other time points. The left panel is representative of rats that displayed signal on day 1, and the right panel representative of rats that did not display a signal on any of the days. Data for the other rats and time points are shown in the ESM. Note that this rat strain can display cycles of thin hair growth, as seen in some images. **f** RARE MRI scans (day 90) of all six rats that received DAPCs (left hemisphere: non-transduced, right hemisphere: Fluc-ZsGreen^+^). No abnormal features are seen, apart from the needle track that is still visible in some animals (indicated arrowheads in the first rat only).

In addition to assessing the ability of BLI and MRI to detect the implanted cells, a further objective of the *in vivo* studies was to investigate whether the presence of either the Fluc-ZsGreen reporter or the MPIOs affected (i) the tumourigenicity of the cells, (ii) the ability of the hESC-derived DAPCs to differentiate into mature DA neurons *in vivo* or (iii) the immunogenicity of the human cells. To this end, three groups of rats were set up. Group 1, which served as a control group for tumour formation, comprised of three rats that had Fluc-ZsGreen^+^ hESCs implanted into the right striatum and unlabelled hESCs into the left striatum (Fig. 2a); group 2 comprised of six rats that had DAPCs derived from Fluc-ZsGreen^+^ hESCs implanted into the right striatum and unlabelled cells into the left striatum (Fig. 2d); group 3 comprised of six rats that had MPIO-labelled hESC-derived DAPCs implanted into the right striatum and unlabelled hESC-derived DAPCs implanted into the left striatum (Fig. 4c).

### hESCs and DAPCs Follow Distinct Fates *In Vivo*, Irrespective of the Introduction of a Reporter Gene

Optical imaging of animals that received undifferentiated hESCs on the administration day and on days 14 and 27 post administration revealed great variability in the bioluminescence signal. On the administration day, just one of the animals displayed a signal, which was very weak, suggesting that Fluc expression was not robust enough for sensitive detection in all animals. Bioluminescence was progressively lost from this rat but detected in a different animal at a later time point (ESM Fig. 2a).

MR imaging of these rats at the experimental endpoint (day 27) displayed a large area of atypical hyperintense contrast surrounding the injection site, which was present in both brain hemispheres of all animals (Fig. 2b and ESM Fig. 2b). Histological analysis of the tissue showed that this area consisted of a large number of tightly packed cells as evidenced by strong nuclear (haematoxylin) staining in the same area (Fig. 2b), suggesting an abnormal growth of cells. Immunofluorescence microscopy of these samples revealed that the masses in both hemispheres consisted of human cells, as evidenced by positive staining for a human-specific nuclear antigen (hNuclei). Interestingly, however, not all cells in the masses that formed in the right brain hemisphere (Fluc-ZsGreen^+^ hESCs) expressed ZsGreen, suggesting that some of the hESCs lost expression of the reporter (Fig. 2c). The hESC-derived masses did not display a teratoma-like tissue architecture when examined by haematoxylin and eosin staining (data not shown). Instead, many of the cells expressed β-III tubulin, suggesting that transplantation of these cells in the rat brain promoted differentiation to ectodermal lineages (ESM Fig. 2c). The growths were also negative for OCT4, confirming that cells had differentiated in the brain (ESM Fig. 2d). Taken together, these results indicate that undifferentiated hESCs form mass lesions, irrespective of the introduction of the Fluc-ZsGreen reporter.

For rats implanted with DAPCs (group 2) (Fig. 2d), four of six animals displayed a bioluminescence signal on the administration day (Fig. 2e and ESM Fig. 3a), which was not detectable at the subsequent imaging points (days 14, 28, 56 and 91). In contrast to hESCs, administration of DAPCs resulted in no abnormal MR contrast at the experimental endpoint (day 91, Fig. 2f), with all animals exhibiting normal brain structures and the needle track being the only remarkable feature.

**Fig. 3.**
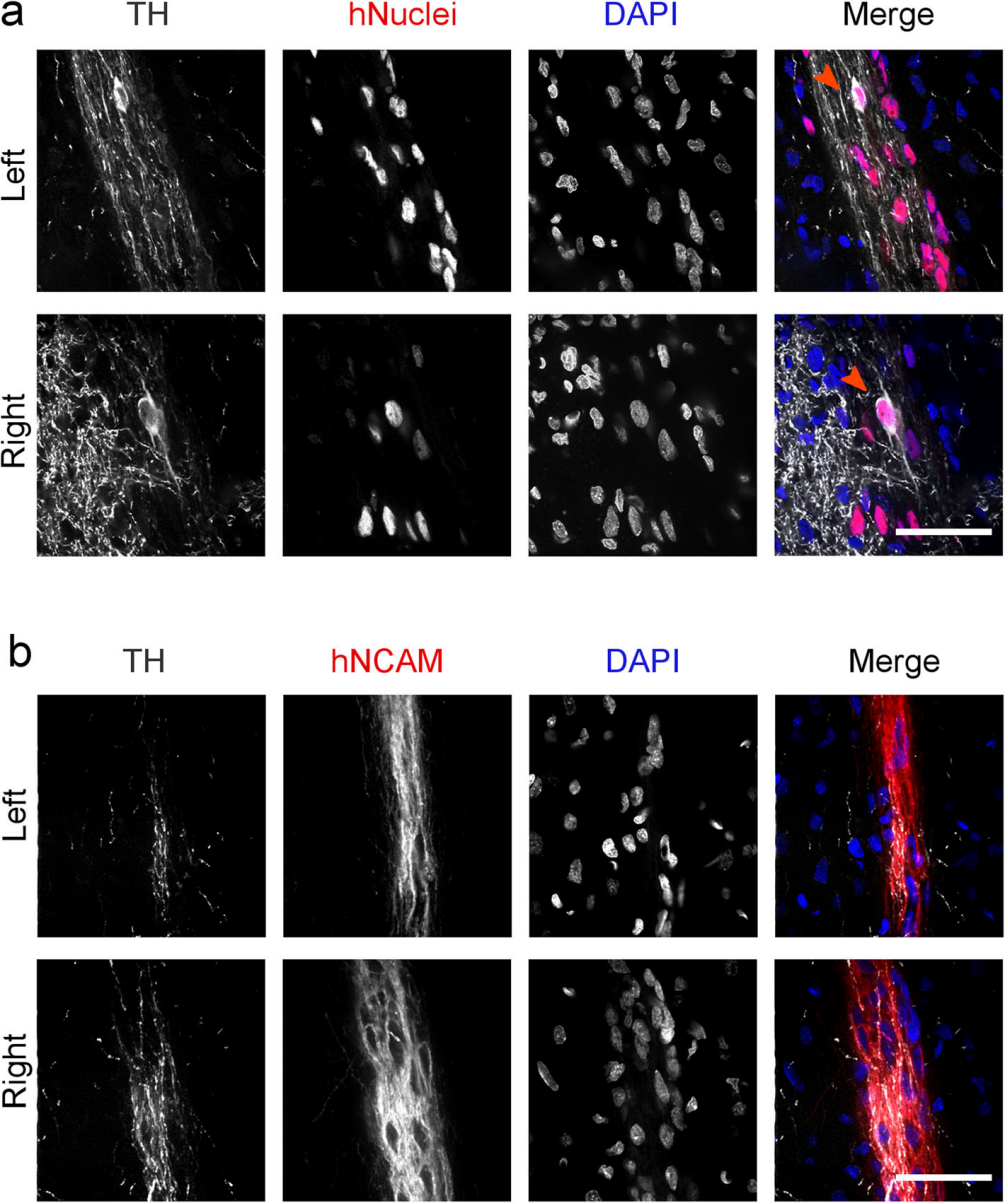
**DAPC integration with the rat brain. a** Immunofluorescence microscopy of the injection sites (left hemisphere: non-transduced, right hemisphere: Fluc-ZsGreen^+^). Cells express a human nuclear antigen, showing that the DAPCs survived in the rats’ brains and expressed TH, suggesting that they matured into DAs. Arrowhead indicates a human cell strongly expressing TH. **b** Immunofluorescence of a similar area but using an antibody against human NCAM as a means to confirm the human origin of the cells. Scale bars correspond to 50 μm.

Human cells were still present at the injection site in both hemispheres, as evidenced by hNuclei positivity (Fig. 3a). Importantly, the areas containing human cells were also positive for TH, suggesting maturation of some DAPCs in the rats’ brains within the experimental period (91 days). Not all human cells robustly expressed TH, likely because a period of >20 weeks is necessary for the maturation of all DAPCs. The injection sites were also positive for a human-specific NCAM (hNCAM) antigen (Fig. 3b), providing further evidence that human cells had integrated with the rat brain and displayed neural lineage commitment, irrespective of whether they had been genetically modified.

### MPIO Labelling Enables Assessment of the Intracranial Distribution of Implanted Cells

**Fig. 4.**
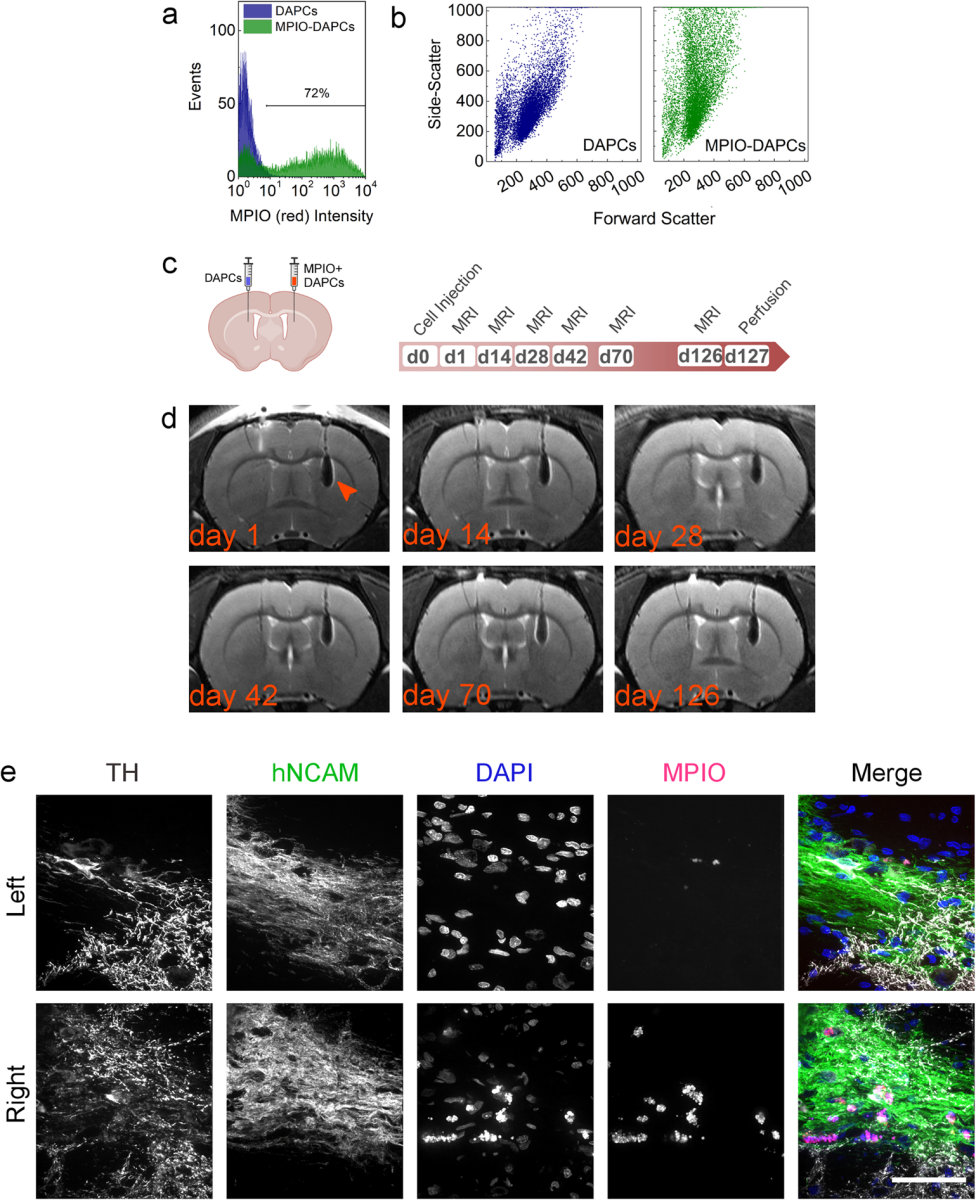
**MPIO tracking of DAPCs in the rat brain. a** Yellow fluorescence of unlabelled and MPIO-labelled DAPCs. **b** Forward *vs.* side scatter plot of unlabelled and MPIO-labelled DAPCs. **c** Schematic of injection and experimental timeline of DAPC administration and magnetic resonance imaging. **d** Representative RARE MRI scans of a rat that received MPIO-labelled DAPC (left hemisphere: unlabelled, right hemisphere: labelled) as imaged on day 1, 14, 28, 42, 70 and 126 post administration. Hypointense contrast, indicative of a reduction in relaxation time as caused by MPIO labelling, is seen in the right hemisphere throughout the experimental period (indicated with an arrowhead in the first image). No abnormal growth is observed in either of the hemispheres. **e** Immunofluorescence microscopy of the injection sites. Cells express human NCAM, showing that MPIO-labelled DAPCs survived in the rats’ brains and TH, suggesting that DAPCs matured into DAs. MPIO-specific fluorescence is only seen in the right hemisphere and tends to be localised near to the cell nuclei. Note that the MPIO fluorophore, Suncoast Yellow, is also excited at 405 nm and bleeds into the DAPI channel. Scale bar = 50 μm.

Flow cytometry analysis of MPIO-labelled DAPCs suggested that approximately 72 % of DAPCs were labelled with the particles, as evidenced by yellow fluorescence originating from MPIOs (Fig. 4a). We also detected a shift in the side scatter of DAPCs after labelling with MPIOs, providing further evidence for the internalisation of the particles (Fig. 4b).

Rats implanted with MPIO-labelled DAPCs (group 3) (Fig. 4c) were imaged only *via* MR as neither of the hemispheres received cells with the genetic reporter. Monitoring of this group for up to 4 months post implantation confirmed that DAPC implantation does not lead to tumour formation, with all rats displaying normal brain structures at all time points. In five out of six rats, hypointense contrast was seen in the right brain hemisphere (Fig. 4d and ESM Fig. 3b). This was an expected consequence of the MPIO labelling, which enabled us to monitor the delivery and intracranial distribution of DAPCs. Remarkably, the distribution of the administered DAPCs appears to remain stable throughout the 4 months that the animals were monitored for, with no obvious change in the area with hypointense contrast, suggesting that the DAPCs were confined to the areas into which they were initially deposited. In one rat, no hypointense contrast was observed in the target area. Further analysis of the scans revealed that for this animal, the needle had been inserted at an angle, with the cells delivered to the ventricle leading to them becoming lodged at a different anatomical location (ESM Fig. 3c).

As observed before, immunofluorescence staining at the injection sites confirmed the presence of human cells that expressed TH, reinforcing the point that these cells were able to integrate within the rat brain and differentiated into mature DAs, irrespective of the MPIO labelling (Fig. 4e). In the right hemisphere, MPIOs were found in the same areas as the administered human cells and appeared to localise to perinuclear regions.

### Intense Staining for GFAP Is Observed Surrounding the Human Cell Implants

**Fig. 5.**
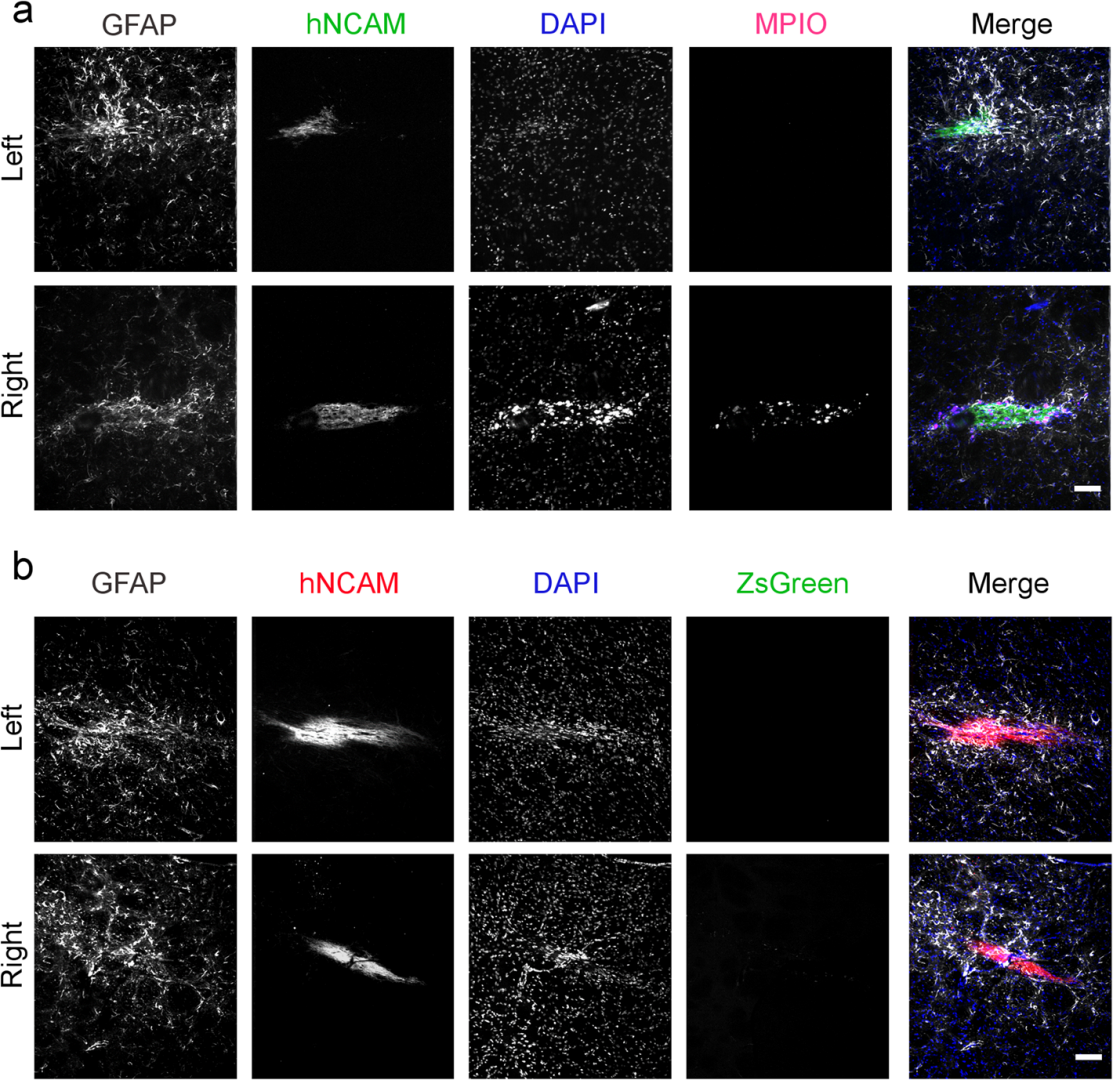
**Glial reaction at the injection sites. a** Immunofluorescence microscopy of brains from rats that received MPIO-labelled DAPCs (left hemisphere: unlabelled; right hemisphere: labelled). The presence of human cells is identified with hNCAM staining, and the intensity of GFAP staining is stronger in these areas. MPIOs are only seen in the right hemisphere. **b** Immunofluorescence microscopy of brains from rats that received Fluc-ZsGreen^+^ DAPCs (left hemisphere: untransduced control cells; right hemisphere Fluc-ZsGreen^+^ cells). Scale bars correspond to 100 μm.

A previous study showed that the implantation of Fluc^+^ hESC-derived neural stem cells into the mouse striatum caused marked glial reaction in the host brain, as evidenced by intense immunostaining for glial fibrillary acidic protein (GFAP), a marker of reactive astrocytes [10]. To investigate whether Fluc-ZsGreen or MPIOs contributed to this reaction, sections from group 2 and group 3 rats were immunostained for GFAP. Qualitative analysis showed an increase in GFAP staining around the human implants, but no differences in staining intensity were observed around the implants comprising unlabelled human cells or MPIO-labelled cells (Fig. 5a and ESM Fig. 4a) and cells derived from Fluc-ZsGreen^+^ hESCs (Fig. 5b and ESM Fig. 4b). Consistent with the expression profile of ZsGreen in mature DA neurons *in vitro* (Fig. 1e), the expression of ZsGreen in the brain sections was barely detectable (Fig. 5b and ESM Fig. 4b).

## Discussion

Our study assessed the effectiveness of BLI and MR imaging to monitor the tumourigenicity, viability and intracranial biodistribution of hESC-derived DAPCs following stereotactic injection into the rat striatum. In most animals, BLI could only detect Fluc-ZsGreen^+^ cells shortly after administration and was not effective for monitoring tumourigenicity and cell viability in the longer term. MR imaging, on the other hand, could detect tumours arising from undifferentiated hESCs and could monitor the intracranial biodistribution of MPIO-labelled hESC-derived DAPCs over the full time-course of our experiments.

The inability to detect cells with BLI likely resulted from a number of factors. First, at the initial imaging session, the rats were only 6 weeks old. During the intervening 2 weeks before the next imaging session, the rats grew considerably and became more pigmented (see ESM Fig. 3a), causing the intensity of the emitted light to be reduced; this likely explains why after day 1, bioluminescence could only be detected in a rat that had developed a large Fluc-ZsGreen^+^ hESC-derived mass (ESM Fig. 2a, rat 3). Bernau *et al.* found that Fluc^+^ human foetal neuronal progenitors implanted into the rat striatum could be imaged with BLI for 3 months [15]. However, they implanted 9 × 10^5^ Fluc^+^ cells into the left hemisphere compared with only 1.5 × 10^5^ cells in our study. An additional problem was that in comparison with undifferentiated hESCs, we found that the expression levels of the reporter genes decreased by ~50 % in Fluc-ZsGreen^+^ hESC-derived DAPCs and could not be detected at all in the mature DA neurons. It is well recognised that ESC differentiation is accompanied by increased levels of DNA methylation, leading to gene silencing, and that the pattern of silencing is cell type specific [16]. The choice of promoter also affects the extent of silencing. A previous study comparing the activity of five constitutive promoters, EF1α, human β-actin (ACTB), cytomegalovirus (CMV), phosphoglycerate kinase (PGK) and ubiquitinC (UbC) in differentiating hESCs, reported that EF1α was the most stable, with expression levels in EBs being reduced to ~50 % of those in undifferentiated hESCs [17]. Our observation that Fluc-ZsGreen expression was undetectable in the mature DA neurons, both *in vitro* and *in vivo*, was unexpected. Tennstaedt *et al.* have shown that a EF1α:Fluc-GFP^+^ neural stem cell line derived from hESCs could be detected with BLI for 6 weeks following injection into the mouse brain without any noticeable decrease in bioluminescence intensity [18]. However, the neural stem cells used in the Tennstaedt study have a different phenotype to hESC-derived DAPCs, and there is no evidence that they differentiate into the DA lineage [18]. Likewise, there is no evidence that the Fluc^+^ human foetal neuron progenitors used in the aforementioned Bernau *et al.* study differentiate into the DA lineage in the rat brain [15]. Indeed, as far as we are aware, there are no studies that show Fluc expression in hESC-derived DA neurons *in vivo* when Fluc is under the control of a constitutive promoter. In future studies, a cell type-specific promoter, such as FOXA2, which is expressed in both DAPCs and mature DA neurons [19], could prove more effective than the EF1α promoter for monitoring viability longitudinally, especially if used with the highly sensitive AkaLuc luciferase in combination with the substrate Akalumine [20]. However, one advantage of our system is that the loss of signal is due to differentiation. This could be used to show that the grafted cells have indeed followed the correct pathway post implantation rather than dedifferentiated back into an ESC-like phenotype.

Four weeks after implantation of undifferentiated hESCs, MR imaging could detect a cell mass in both cerebral hemispheres, irrespective of whether the cells had been transduced with the Fluc-ZsGreen reporter (ESM Fig. 2b). However, no cell masses were detected at any time point following administration of hESC-derived DAPCs, suggesting that in contrast to the undifferentiated hESCs, the DAPCs are non-tumourigenic. Cells labelled with MPIOs could be detected at all time points using longitudinal MR imaging. In addition, we found that the cells remained at the injection site and did not appear to migrate to other brain regions. From a safety perspective, the lack of migration is important to prevent cells integrating into intact neural circuits causing side effects (*e.g.* epilepsy) [21].

Previous studies have shown that labelling cells with iron oxide nanoparticles can inhibit differentiation to specific lineages [22, 23]. In our study, we did not find any evidence that the bicistronic Fluc-ZsGreen reporter or the MPIOs inhibited the differentiation of hESC-derived DAPCs into TH^+^ DA neurons. The final aim of our study was to investigate whether the reporter or the MPIOs increased glial reactivity to the grafted cells. It is known that the implantation of cells into the brain induces a glial response [24], as evidenced by increased numbers of reactive GFAP^+^ astrocytes surrounding the grafts [10]. Transplantation of both labelled and unlabelled DAPCs elicited a marked glial reaction at the injection site, as expected. However, there was no notable difference in the scale of glial response, suggesting that neither Fluc-ZsGreen nor MPIOs increased the glial reaction to the DAPCs [25].

## Conclusions

In summary, we have demonstrated that hESC-derived DAPCs can be labelled with luminescence and contrast-enhancing reporters for *in vivo* cell tracking. Following intracranial transplantation in the rat striatum, our findings support the safe implementation of DAPC-derived therapies for the treatment of PD.

## Electronic supplementary material

ESM 1 (DOCX 8064 kb)
