# Supplementary material for: Assessing Human Embryonic Stem Cell-Derived Dopaminergic Neuron Progenitor Transplants Using Non-invasive Imaging Techniques
Source: Mol Imaging Biol. 2020 May 6;22(5):1244–54. doi: 10.1007/s11307-020-01499-4 (PMC7497430; doi:10.1007/s11307-020-01499-4)
Supplement: Supplementary file 1 — (DOCX 8064 kb) [file 11307_2020_1499_MOESM1_ESM.docx]

**Electronic Supplementary Information**

**Assessing human embryonic stem cell-derived dopaminergic neuron progenitor transplants using non-invasive imaging techniques**

**Masoumeh Mousavinejad^1^, Sophie Skidmore^1, 3^, Francesco G. Barone^1^, Pamela Tyers^2^, , Venkat Pisupati^3^, Harish Poptani^1^, Antonius Plagge^1^, Roger A. Barker^2, 3^, Patricia Murray^1^, Arthur Taylor^1, 5^ & Christopher J. Hill^1, 4, 5^**

**Author information**

^1^Department of Cellular and Molecular Physiology, Institute of Translational Medicine, University of Liverpool, Liverpool L69 3BX, UK

^2^John van Geest Centre for Brain Repair & Department of Neurology, Department of Clinical Neurosciences, University of Cambridge, Cambridge, UK

^3^WT-MRC Cambridge Stem Cell Institute, University of Cambridge, Cambridge, UK

^4^Centre for Women’s Health Research, Department of Women’s and Children’s Health, Institute of Translational Medicine, University of Liverpool, Liverpool L8 7SS, UK

^5^Joint corresponding authors

To whom correspondence should be addressed:

Dr Arthur Taylor

Email: taylora@liverpool.ac.uk

Telephone: 0151 795 4456

Dr Christopher Hill

Email: C.J.Hill1@liverpool.ac.uk

Telephone: 0151 795 9584

**
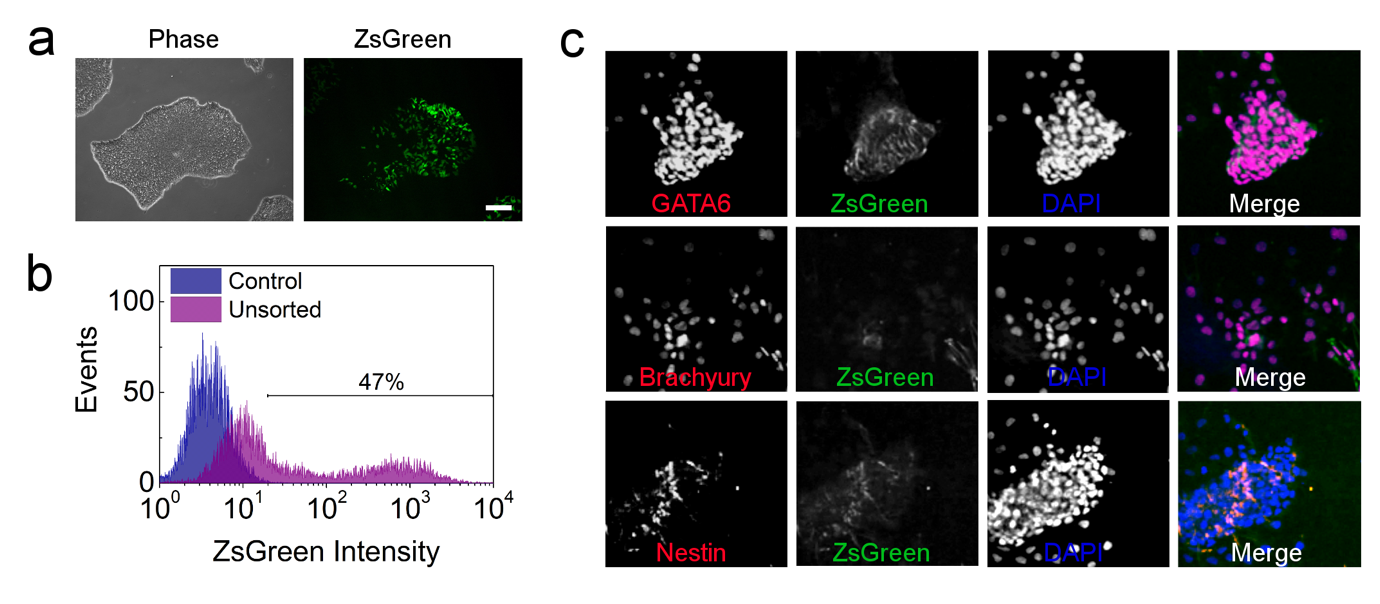
**

**ESM Fig 1. (a)** Phase and fluorescence microscopy images of hESCs transduced with the FLuc-ZsGreen vector. Not all of the cells express the transgene. Scale bar = 100 μm. **(b)** Flow cytometry of the same cells showing that prior to sorting, only 47% of the cells expressed the transgene. The cells underwent cell sorting to obtain a pure homogeneous population with strong expression of the reporters. **(c)** Embryoid bodies formed from sorted hESCs were shown to be able to differentiate into all three germ layers, as evidenced by positive staining for GATA6 (endoderm), brachyury (mesoderm) and nestin (ectoderm). To generate embryoid bodies, undifferentiated RC17 cells were treated with Accutase (Stem Cell Technologies) to generate a single cell suspension. 10^6^ cells were suspended in APEL 2 medium (Stem Cell Technologies) supplemented with 10 μM Y-27632 and added into a well of an AggreWell800 plate (Stem Cell Technologies). AggreWell plates were centrifuged to deposit the cells and incubated at 37°C with 5% CO_2_. Medium was changed every 2 days until day 10. For immunocytochemical analysis of germ layer markers, EBs were transferred to Matrigel (Corning) coated cell culture plates for a further 10 days.


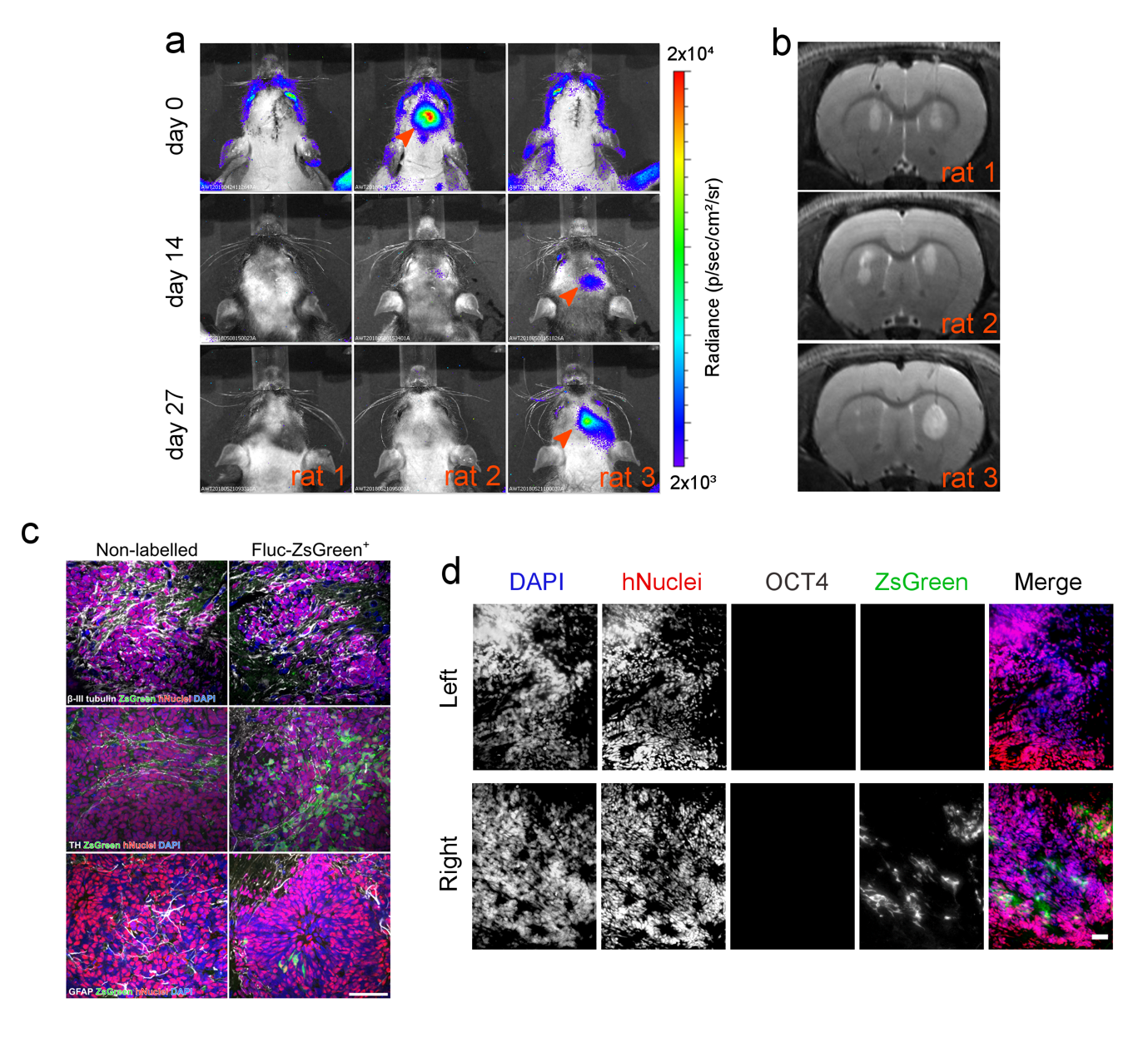


**ESM Fig 2. (a)** BLI of rats that received undifferentiated hESCs (left hemisphere: non-transduced, right hemisphere Fluc-ZsGreen^+^). On the administration day (day 0) rat 2 displayed a signal, which was weak and close to background (arrowhead). This signal was lost on the subsequent measurement days. Rat 3 displayed a signal only on days 14 and 27 (arrowheads). Due to the weak signal some non-specific background can be seen in the outlines on the rats’ head on day 0. **(b)** Corresponding MRI of the brain on day 27. All rats developed tumours (hyperintense contrast) of varying sizes in both hemispheres. MRI of rat 1 is displayed in the main manuscript and is duplicated here to facilitate comparison with the other conditions. **(c)** Representative micrographs of tumours stained for human nuclei and ectodermal markers (β-III tubulin, tyrosine hydroxylase or GFAP). A subpopulation of cells expressing ZsGreen can be observed in the tumours formed from Fluc-ZsGreen^+^ hESCs. Autofluorescence in the green channel from the rat striatum is responsible for the weak signal in the left hemisphere. Scale bar = 50 μm **(d)** Tumour cells are positive for human nuclei but negative for OCT4, confirming that all injected hESCs differentiated to some extent in the rat brain. Scale bar = 100 μm.

**
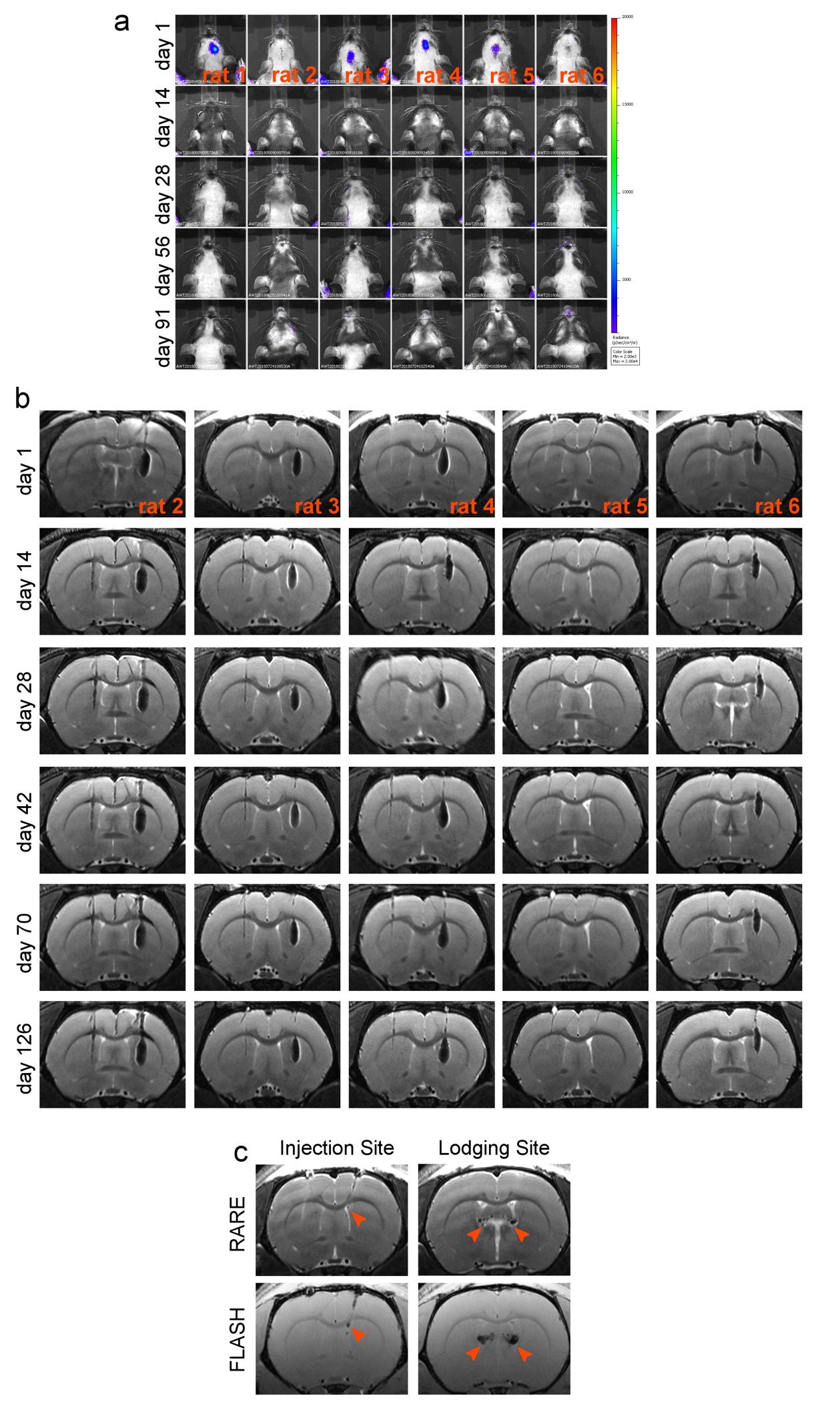
**

**ESM Fig 3. (a)** Bioluminescence imaging of the head of rats that received DAPCs. Rats were imaged on the administration day (day 0) and at day 1, 14, 28, 56 and 91. Although bioluminescence signal was detected in 4 of 6 rats on the administration day, this signal was lost in the subsequent days. Data from rats 2 and 4 are shown in the main manuscript and reproduced here to facilitate comparison. **(b)** RARE MRI scans of rats that received MPIOs labelled DAPC (left hemisphere: unlabelled, right hemisphere: labelled) as imaged on day 1, 14, 28, 42, 70 and 126 post surgery. Hypointense contrast, indicative of a reduction in relaxation time as caused by MPIO labelling, in seen in the right hemisphere throughout the experimental period. Data from rat 1 is shown in the main manuscript. **(c)** Day 1 RARE and FLASH MRI imaging of rat 5, where MPIO-labelled DAPCs were misinjected. Left: The needle track is clearly identified in the MR images, revealing an injection angle that led to cells being administered to the ventricle (arrow heads). Right: imaging of a different plane reveals hypointense contrast in a different area within the ventricles (arrowheads), indicating that the cells migrated and lodged in an unintended site. FLASH images are shown as they provide better contrast between the brain and MPIOs, facilitating the identification of the needle track. Note that hypointense contrast is seen in both hemispheres of the brain of the rat in which cells were misinjected, which is likely due to the bridged nature of the fluid-filled ventricles. FLASH acquisition parameters: TE = 5.5 ms, TR = 265 ms, flip angle = 20o, NEX = 5, field of view (FOV) = 35x35 mm, matrix size = 350x350 pixels, slices = 20, slice thickness = 500 µm.


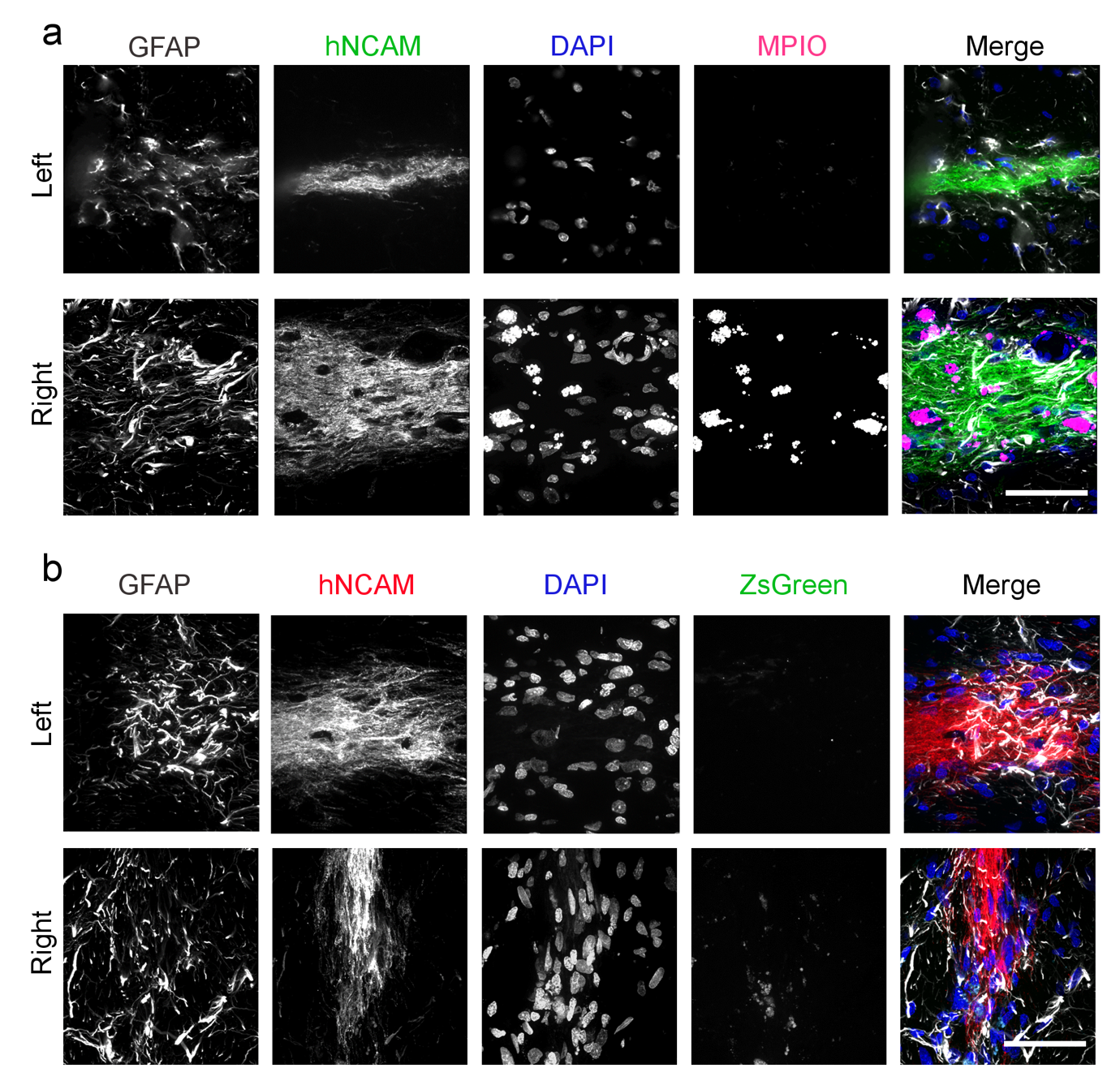

**ESM Fig 4.** High power images of glial cell reaction at the injection sites post DAPC administration. **(a)** Immunofluorescence microscopy of brains from rats that received MPIO-labelled DAPCs (left hemisphere: unlabelled; right hemisphere: labelled). The presence of human cells is identified with hNCAM staining, and the intensity of GFAP staining is stronger in these areas. MPIOs are only seen in the right hemisphere. **(b)** Immunofluorescence microscopy of brains from rats that received Fluc-ZsGreen+ DAPCs (left hemisphere - untransduced control cells; right hemisphere - Fluc-ZsGreen+ cells). Scale bars = 100 µm.

**ESM Table 1.** Primer sequences used for RT-qPCR

| **Gene** | **Full gene name** | **Primer sequence 5’ - 3’ (fwd/rev)** |
| --- | --- | --- |
| **GAPDH** | Glyceraldehyde-3-phosphate dehydrogenase | GTGGAAGGACTCA TGACCA  GAGGCAGGGATG ATGTTCT |
| **FOXA2** | Forkhead box A2 | CCGTTCTCCATCAACAACCT  GGGGTAGTGCATCACCTGTT |
| **OTX2** | Orthodenticle homeobox 2 | ACAAGTGGCCAATTCACTCC  GAGGTGGACAAGGGATCTGA |
| **LMX1A** | LIM homeobox transcription factor 1 alpha | CGCATCGTTTCTTCTCCTCT  CAGACAGACTTGGGGCTCAC |

**ESM Table 2.** Antibodies used for immunofluorescence

| **Marker** | **Species** | **Dilution** | **Company** | **Catalogue Number** | **Secondary Antibody** |
| --- | --- | --- | --- | --- | --- |
| **β-III tubulin** | Monoclonal Mouse IgG2A | 1:1000 | Biolegend | 801201 | AlexaFluor 488 Goat anti Mouse IgG2A |
| **Brachyury** | Polyclonal Goat IgG(H+L) | 1:500 | Santa Cruz Biotechnology | sc-17743 | AlexaFluor 647 Donkey anti Goat IgG(H+L) |
| **FOXA2** | Polyclonal Goat IgG(H+L) | 1:200 | SantaCruz Biotechnology | sc-655 | AlexaFluor 594 Donkey anti Goat IgG(H+L) |
| **GATA6** | Polyclonal Rabbit IgG(H+L) | 1:500 | Santa Cruz Biotechnology | sc-9055 | AlexaFluor 594 Chicken anti Rabbit IgG(H+L) |
| **GFAP** | Polyclonal Rabbit IgG(H+L) | 1:1000 | DAKO | ZO334 | AlexaFluor 647 Chicken anti Rabbit IgG(H+L) |
| **Human NCAM** | Monoclonal Mouse IgG1 | 1:100 | Santa Cruz Biotechnology | Sc-106 | AlexaFluor 488/594 Goat anti Mouse IgG1 |
| **Human nuclei** | Monoclonal Mouse IgG1 | 1:200 | Merck | MAB1281 | AlexaFluor 594 Goat anti Mouse IgG1  HRP conjugated Goat anti Mouse IgG(H+L) |
| **LMX1A** | Polyclonal Rabbit IgG(H+L) | 1:500 | Abcam | Ab139726 | AlexaFluor 595 Chicken anti Rabbit IgG(H+L) |
| **Nestin** | Polyclonal Rabbit IgG(H+L) | 1:250 | Abcam | ab92391 | AlexaFluor 594 Chicken anti Rabbit IgG(H+L) |
| **OCT4** | Monoclonal Mouse IgG2b | 1:500 | Santa Cruz Biotechnology | sc-5279 | AlexaFluor 647 Goat anti Mouse IgG2b |
| **OTX2** | Polyclonal Goat IgG(H+L) | 1:500 | R&D Systems | AF-1979 | AlexaFluor 594 Donkey anti Goat IgG(H+L) |
| **Tyrosine hydroxylase** | Polyclonal Rabbit IgG(H+L) | 1:1000 | Merck | AB152 | AlexaFluor 647 Chicken anti Rabbit IgG(H+L) |
